# Effects of Different Soil Phosphorus Levels on the Physiological and Growth Characteristics of *Phyllostachys edulis* (Moso Bamboo) Seedlings

**DOI:** 10.3390/plants14162473

**Published:** 2025-08-09

**Authors:** Zhenya Yang, Benzhi Zhou

**Affiliations:** 1 Zhejiang Academy of Forestry, Hangzhou 310023, China; yangzhenya1234@163.com; 2 Northwest Zhejiang Bamboo Forest Ecosystem Positioning Observation and Research Station, National Forestry and Grassland Administration, Hangzhou 310023, China; 3 Research Institute of Subtropical Forestry, Chinese Academy of Forestry, Hangzhou 311400, China

**Keywords:** phosphorus addition, root architecture, nutrient element allocation, non-structural carbohydrates

## Abstract

Soil phosphorus (P) availability is a critical factor affecting the productivity of *Phyllostachys edulis* (moso bamboo) forests. However, the mechanisms underlying the physiological and growth responses of moso bamboo to varying soil P conditions remain poorly understood. The aim of this study was to elucidate the adaptive mechanisms of moso bamboo to different soil P levels from the perspectives of root morphological and architectural plasticity, as well as the allocation strategies of nutrient elements and photosynthates. One-year-old potted seedlings of moso bamboo were subjected to four P addition treatments (P1: 0, P2: 25 mg·kg^−1^, P3: 50 mg·kg^−1^, P4: 100 mg·kg^−1^) for one year. The biomass of different seedling organs, root morphological and architectural indices, and the contents of nitrogen (N), P, and non-structural carbohydrates in the roots, stems, and leaves were measured in July and December. P addition increased the root length (by 113.8%), root surface area (by 146.5%), root average diameter (by 14.8%), root length ratio of thicker roots (diameter > 0.9 mm), number of root tips (by 31.9%), fractal dimension (by 5.6%), P accumulation (by 235.8%), and contents of starch (ST) and soluble sugars (SS), while it decreased the specific root length (by 31.7%), root branching angle (by 1.9%), root topological index (by 4.8%), root length ratio of finer roots (diameter ≤ 0.3 mm), SS/ST, and N/P. The root–shoot ratio showed a downward trend in July and an upward trend in December. Our results indicated that moso bamboo seedlings tended to form roots with a small diameter, high absorption efficiency, and minimal internal competition to adapt to soil P deficiency and carbon limitation caused by low P. Under low-P conditions, moso bamboo prioritized allocating photosynthates and P to roots, promoting the conversion of starch to soluble sugars to support root morphological and architectural plasticity and maintain root growth and physiological functions. Sole P addition eliminated the constraints of low P on moso bamboo growth and nutrient accumulation but caused imbalances in the N/P.

## 1. Introduction

Moso bamboo (*Phyllostachys edulis* (Carr.) H. de Lehaie f. edulis) is a monopodial scattered evergreen arborescent bamboo species. Moso bamboo forests exhibit the largest distribution area in China, with both bamboo shoot and timber yields ranking first nationwide. Consequently, moso bamboo has become the most economically valuable bamboo species in the country [1]. Phosphorus (P) is an essential macronutrient for plant growth and development. As a critical component of macromolecules such as nucleic acids, phospholipids, and enzymes, it directly influences fundamental metabolic processes in plants, including photosynthesis, respiration, signal transduction, nucleic acid metabolism, and lipid metabolism [2]. Although the total P content in the soil is high, most of it exists in the form of organic phosphorus that plants cannot directly utilize. Approximately 70% to 90% of the remaining inorganic P is fixed by the soil, resulting in an extremely low content of available P in the soil [3]. In subtropical regions of China, soil available P deficiency is particularly severe, emerging as a critical limiting factor for enhancing forest productivity—notably in moso bamboo forests [4,5]. To address soil P deficiency, phosphorus fertilization is widely practiced to meet plant P requirements. However, inefficient and excessive P fertilizer use not only elevates forestry production costs but also triggers ecological issues, such as soil eutrophication. Thus, clarifying the adaptation mechanisms of moso bamboo to varying soil P conditions serves as a prerequisite for optimizing P supply strategies in moso bamboo forest management.

To adapt to heterogeneous soil P conditions, plant roots typically exhibit morphological, architectural, and physiological plasticity. Morphologically, under low-P stress, plants display species-specific root system modifications, such as taproot shortening, root diameter reduction, increased root length density, promotion of root hair proliferation and elongation, and development of aerenchyma tissues [6,7,8]. In terms of root architecture, low-P soil conditions induce plants to increase root branching angles, forming an “umbrella-shaped” root system architecture that enhances P acquisition from surface soil. Alternatively, plants may develop cluster roots through lateral root proliferation, thereby expanding the absorption zone and enhancing the secretion of P-mobilizing compounds (e.g., organic acids) [9,10]. These modifications in root morphology and architecture enable plants to allocate limited carbohydrates toward forming a larger root absorption surface area, thereby enhancing P acquisition. However, existing studies merely infer changes in root architecture by analyzing root density and distribution across soil layers. Root architectural plasticity in plants under different soil P conditions, such as root branching direction, intensity, and patterns, remains a significant research gap. These key aspects of root architectural plasticity have not been quantified using indices such as root branching angles, topological index, and fractal dimension [11,12]. These unaddressed aspects are crucial for deeply elucidating plant root foraging preferences toward phosphorus patches under different P levels, as well as the carbon investment trade-offs between taproots and lateral roots. For moso bamboo, conclusions regarding its growth responses to changes in soil P availability are not consistent. For example, a study has found that P addition increases the root biomass of moso bamboo by 21.5%, while contradictory results indicate that low P enhances root length by 29.5% and root surface area by 13.6% [13,14]. Notably, moso bamboo exhibits stronger P foraging ability than the typical subtropical tree species *Pinus massoniana* Lamb. and *Cunninghamia lanceolata* (Lamb.) Hook., as evidenced by its higher root length response ratio (0.157) to high-P patches compared with *P. massoniana* (0.103) and *C. lanceolata* (0.139). Moso bamboo can also directionally transport photosynthates to roots, promoting root growth and thickening in phosphorus-enriched soil patches [15]. However, none of these studies have addressed the responses of root morphology and architecture in moso bamboo to varying soil P levels.

When resource acquisition is constrained, plants with strong adaptability preferentially allocate a higher proportion of limited nutrient elements and photosynthates to functionally active organs undergoing rapid growth, such as root tips, young leaves, and shoot apical meristems [16,17]. This is done to maximize the functional benefits of these organs and, to the greatest extent possible, to maintain the basic physiological growth requirements. For instance, under low-P conditions, the upregulated expression of plant sucrose transporter genes redirects more sucrose to roots for mycorrhizal symbiosis or cluster root formation, thereby enhancing the phosphorus absorption capacity. *C*. *lanceolata* allocates more P to leaves to maintain photosynthetic function under low-P conditions [18,19]. Elucidating the allocation patterns of nutrients and photosynthates can profoundly clarify the trade-off strategies of plants between carbon cost investment (carbon consumption) and resource benefits (nutrient acquisition and carbon fixation), which also represents one of the core mechanisms for their adaptation to diverse P environments globally. Previous studies have shown that bamboo can adapt to low P by regulating biomass, nitrogen and P allocation patterns, and hormone levels in different organs [13,20]. However, the adaptive mechanisms of moso bamboo under multi-gradient phosphorus levels, the allocation patterns of photosynthates, and the relationship between photosynthate allocation patterns and root morphological plasticity have not been fully investigated.

Although the effects of soil phosphorus variation on plant growth are significant and multifaceted, the numerous confounding variables between soil phosphorus and plants may compromise our judgment of phosphorus impacts, including plant age, soil pH, organic matter, microorganisms, mineral element contents, and soil texture [21,22]. Therefore, it is necessary to control P addition as a single variable to minimize the influence of confounding variables. The response strategies of plants to different soil P conditions (including growth, root morphology, resource allocation patterns, etc.) are not consistent, as they are influenced by multiple factors, including plant adaptability, developmental stages, and the duration and intensity of P stress [23,24]. For example, plants with shallow root systems and stronger organic acid secretion capacity, or nitrogen-fixing plants, are less susceptible to low-P stress [25]. Therefore, studies on the effects of P on plants should include multiple P levels and multiple plant developmental stages.

As highlighted above, existing studies on the effects of soil phosphorus on moso bamboo lack analyses of root architectural traits across different growth stages, thereby precluding researchers from quantifying the strategic transition mechanisms of root architecture under varying P levels. Additionally, the absence of investigations into carbon–photosynthate allocation strategies hinders the clarification of how moso bamboo balances carbon investment among different functional organs. The objective of this study is to dissect the adaptive mechanisms of moso bamboo to diverse soil P conditions by comprehensively investigating biomass accumulation, morphological plasticity, nutrient allocation, and carbon–photosynthate allocation strategies at distinct developmental stages under varying soil phosphorus gradients. This study posits the following hypotheses: (1) moso bamboo seedlings adapt to low-P conditions by forming roots with lower construction costs and higher absorption efficiency; (2) P addition alleviates low-P inhibition of plant growth and reverses root morphological and architectural plasticity induced by P stress; and (3) moso bamboo employs distinct nutrient and carbon–photosynthate allocation strategies under varying P levels to promote preferential organ growth, with these strategies potentially adjusting throughout the growth and developmental process. Addressing these research gaps will not only deepen the theoretical understanding of plant–soil interactions but also provide a technical framework for “root architecture breeding + precision phosphorus fertilization” in the sustainable management of moso bamboo forests in subtropical low-P red soil regions.

## 2. Materials and Methods

### 2.1. Experimental Set Up

This experiment was carried out at the Research Institute of Subtropical Forestry, Chinese Academy of Forestry, located at 73 Daqiao Road, Fuyang District, Hangzhou City, Zhejiang Province (119°57′ E, 29°48′ N). The location is characterized by a typical subtropical monsoon climate, boasting a frost-free period of 307 days. It has an average annual sunshine duration of 1663.2 h. Additionally, the average relative humidity was 70.3%. The average temperature throughout the test period was 25.3 °C. The experimental soil was collected from the infertile and low-phosphorus soil in moso bamboo forests of Miaoshanwu Experimental Forest Farm, Fuyang District, Hangzhou City, Zhejiang Province. The soil sample (with a pH value of 4.91) had the following characteristics: per kilogram, the soil contained 18.7 g of organic carbon, 0.86 g of total nitrogen, 0.26 g of total phosphorus, 11.2 g of total potassium, 85.13 mg of hydrolyzable nitrogen (AN), 4.15 mg of available phosphorus (AP), and 65.73 mg of available potassium. The soil was collected from moso bamboo forests in subtropical regions of China, aiming to replicate the natural growth conditions of bamboo.

The moso bamboo seedlings used in the experiment were cultivated from the seeds of moso bamboo sourced from the same provenance. The parent bamboo plants were sourced from the forestry center in Dajing Town, Guilin City, Guangxi Zhuang Autonomous Region. Representative seeds were placed on moist filter paper and wetted with deionized water. They were then incubated in an incubator maintained at 25 °C until germination occurred. Seedlings exhibiting radicles of approximately the same length were transplanted into a seedling tray. Once the leaves had emerged, bamboo seedlings with comparable height (7.4 cm) and basal diameter (0.83 mm) were carefully planted into plastic pots with dimensions of 25 × 27 cm. Each plastic pot was filled with 6 kg of soil. The soil moisture was precisely regulated by integrating the soil weighing methodology and a sophisticated soil moisture monitoring system (IMKO GmbH, Trime-pico AZS-100, Ettlingen, Germany). Subsequently, it was consistently maintained at a level corresponding to 80–85% of the maximum field water-holding capacity.

Forty seedlings were divided into four groups, each receiving one of four different dosages of phosphate fertilizer (Na_2_HPO_4_-H_2_O) treatments: 0 (P1), 25 mg·kg^−1^ (P2), 50 mg·kg^−1^ (P3), and 100 mg·kg^−1^ (P4), with 10 seedlings per group. P1 was designated as the control group. According to the nutrient grading standard of the National Soil Census [26], the soil without any fertilizer added (P1) was classified as being in the phosphorus-deficient grade. Based on previous preliminary experiments, it was found that when phosphate fertilizers were applied at dosages of 25 mg·kg^−1^, 50 mg·kg^−1^, and 100 mg·kg^−1^, the soil P could reach the grades of moderate, relatively rich, and rich, respectively. Soil HN, AP, and pH after P addition are shown in Table 1 (measured after two samplings in July and December). After the fertilizer was dissolved in clean water, it was sprayed onto the soil in the pots in three installments, with an interval of 15 days between each fertilization. Consistent lighting and temperature conditions were maintained in the greenhouse. The temperature range in the greenhouse throughout the trial period was 20–35 °C during the daytime and 14–25 °C at night. The greenhouse lighting relied on natural light with a light transmittance of 80%.

### 2.2. Harvest and Measurements

In July and December of the same year, sampling was conducted for 10 seedlings in each treatment, with 5 seedlings sampled each time as 5 replicates. The leaves, stems, and roots were separated using scissors. Subsequently, the soil was carefully shaken to prevent any damage to the root tissues and the root architecture. All residual roots were then collected through a 2 mm sieve. The roots, stems, and leaves were sealed in self-sealing bags, placed in an ice box (at a temperature of 0–2 °C), and then transported back to the laboratory. The roots were meticulously rinsed with water and gently blotted dry using absorbent paper. Subsequently, the roots were scanned with a double-sided scanner at a high resolution of 500 dpi (Regent Instruments Inc., WinRhizo Pro, Québec, QC, Canada). The root images were then analyzed with the WinRhizo software (version 2.0) to accurately determine root morphology parameters, including root length (RL, cm), root surface area (RSA, cm^2^), root average diameter (RD, mm), number of root tips (RT, pieces·plant^−1^), and number of root links, and the root architecture parameters such as the fractal dimension (FD) and root branching angle (RBA, °) were determined (Table 2). The fractal dimension (FD) was calculated by the WinRhizo software using the box-counting method [27].

**Figure 1:**
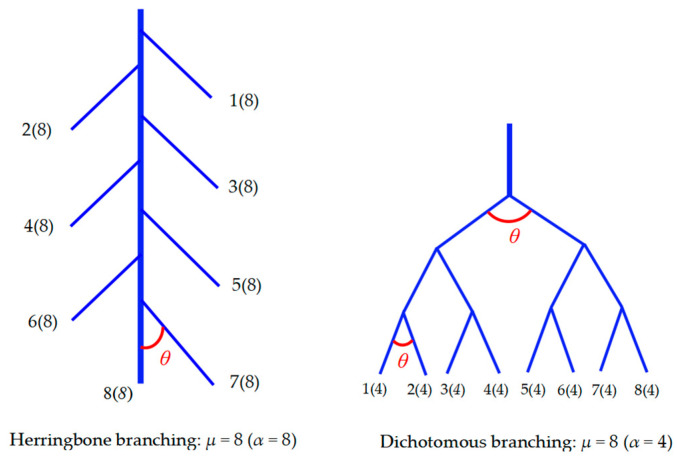
Diagram of root topology classification.

The ratio of the root length of each diameter grade (rl) to the total root length was employed to compute the root length ratio (RLR). Figure 1 denotes a classic classification of root topology.

TI *=* lg (α)/lg (µ)

where TI is the root topological index. The root tips were regarded as external nodes, while the branch points were considered as internal nodes. Correspondingly, the root segments situated between any two of these nodes were defined as links. Links that do not end at a terminal point within the root organizational structure are defined as internal links, while the remaining ones are classified as external links. The external links are classified as external–external (EE) when external links extend from other external links and external–internal (EI) when external links extend from internal links [11,27]. The altitude (α) is defined as the number of internal links along the longest pathway from the root collar to an external root tip. Meanwhile, the magnitude (µ) represents the total quantity of external links within the root system, which is equivalent to the RT [27].

Following the scanning process, the roots, stems, and leaves were immediately heat-killed at 105 °C for 30 min to achieve rapid inactivation of both microbial activity and enzymatic activity, thereby halting post-harvest nutrient transformation and non-structural carbohydrate degradation. Subsequently, these samples were dried at a constant temperature of 65 °C until a constant weight was achieved, enabling the determination of the dry weight (biomass) of the respective tissues. The specific root length (SRL) represents the ratio of the total root length to the dry weight of the roots. After grinding the samples using a high-throughput tissue grinder (Retsch GmbH, MM400, Haan, Germany), the nitrogen (N) contents in the roots, stems, and leaves were determined using the H_2_O_2_-H_2_SO_4_ digestion method [28]. A standard addition recovery experiment was performed using (NH_4_)_2_SO_4_ standard (N content: 21.20%), yielding a N recovery rate of 97.2 ± 0.5% across six replicate measurements. The P contents were measured using Vanadium-Molybdenum Yellow colorimetry, with a 50 μmol/L KH_2_PO_4_ standard solution (prepared from dried KH_2_PO_4_, ≥99.9% purity) yielding a phosphorus recovery rate of 98.2 ± 0.6% (n = 6) [29]. Meanwhile, the concentrations of non-structural carbohydrates [NSCs, i.e., soluble sugar (SS) and starch (ST)], were determined using the anthrone colorimetric method [30].

### 2.3. Statistical Analysis

The significance of differences and correlations among parameters were analyzed by employing SPSS software (version 20.0; SPSS Inc., Chicago, IL, USA). The results were analyzed by one-way ANOVA, followed by Tukey-adjusted post hoc tests that were applied to identify significant differences between groups. Pearson’s correlation analysis method was used to analyze the correlations among indicators. The normality of residuals was statistically tested by the Shapiro–Wilk test. For non-normally distributed data (*p* < 0.05), the following transformation procedures were applied: logarithmic transformation (log10) for right-skewed data, square root transformation for count data, and inverse transformation for reciprocal relationships. Normality was rechecked after transformation, and non-parametric tests (Kruskal–Wallis test or Mann–Whitney U test) were used if normality assumptions were not met.

## 3. Results

### 3.1. Effects of Different Soil Phosphorus Levels on the Biomass Allocation of Moso Bamboo Seedlings

**Figure 2:**
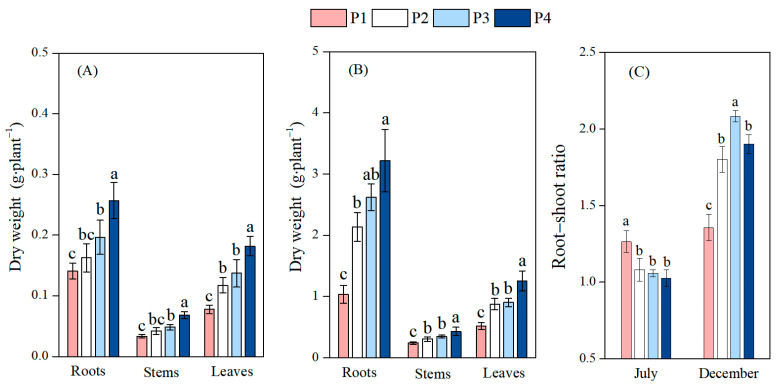
Effects of different soil phosphorus levels on the biomass allocation of moso bamboo seedlings. Note: a, b, c—different letters indicate significant differences among different treatments (*p* < 0.05). The values in the bar chart are the means and standard errors. (**A**): Results from July sampling; (**B**): results from December sampling; (**C**): root–shoot ratio. Four phosphorus addition treatments: P1 (0 mg·kg^−1^), P2 (25 mg·kg^−1^), P3 (50 mg·kg^−1^), and P4 (100 mg·kg^−1^).

The biomass of leaves, roots, and stems and the root–shoot ratio were measured to reflect growth characteristics and carbon allocation strategies of moso bamboo under different soil P levels (Figure 2). As the soil P content gradually increased, the biomass of the roots, stems, and leaves all exhibited a trend of a gradual increase. In the first phase (July), compared with the low-P treatment (P1), P3 significantly increased the biomass of roots, stems, and leaves by 39.5% (*p* < 0.05, 95% CI: 0.011, 0.100 g), 44.6% (*p* < 0.01, 95% CI: 0.006, 0.024 g), and 76.3% (*p* < 0.01, 95% CI: 0.032, 0.087 g), respectively. Similarly, P4 also significantly enhanced the root, stem, and leaf biomass by 82.4% (*p* < 0.01, 95% CI: 0.072, 0.160 g), 100.3% (*p* < 0.01, 95% CI: 0.025, 0.044 g), and 133.9% (*p* < 0.01, 95% CI: 0.077, 0.132 g), respectively, compared to P1. In contrast, the P2 treatment only increased the biomass of leaves by 40.9% (*p* < 0.01, 95% CI: 0.04, 0.059 g). This part of the results supports part of our Hypothesis 2, namely, that P addition alleviates the inhibitory effect of low P on the growth of moso bamboo. All P2, P3, and P4 treatments significantly reduced the root–shoot ratio (*p* < 0.01), with decreases of 14.5%, 16.3%, and 18.9%, respectively. In the second phase (December), compared with the P1 treatment, the P addition treatments (P2, P3, and P4) significantly increased the biomass of roots (by 106.4%, 152.9%, and 210.8%, respectively), stems (by 125.5%, 143.5%, and 178.2%, respectively), and leaves (by 170.0%, 175.1%, and 242.3%, respectively), as well as the root–shoot ratio (by 32.8%, 53.7%, and 40.2%, respectively) (*p* < 0.05). The root–shoot ratio exhibited completely opposite response trends to P addition in the two periods, indicating that the resource allocation strategy of moso bamboo may be adjusted along with the growth and developmental process, which supports part of the views in Hypothesis 3.

### 3.2. Effects of Different Soil Phosphorus Levels on the Root Morphology and Architecture of Moso Bamboo Seedlings

**Figure 3:**
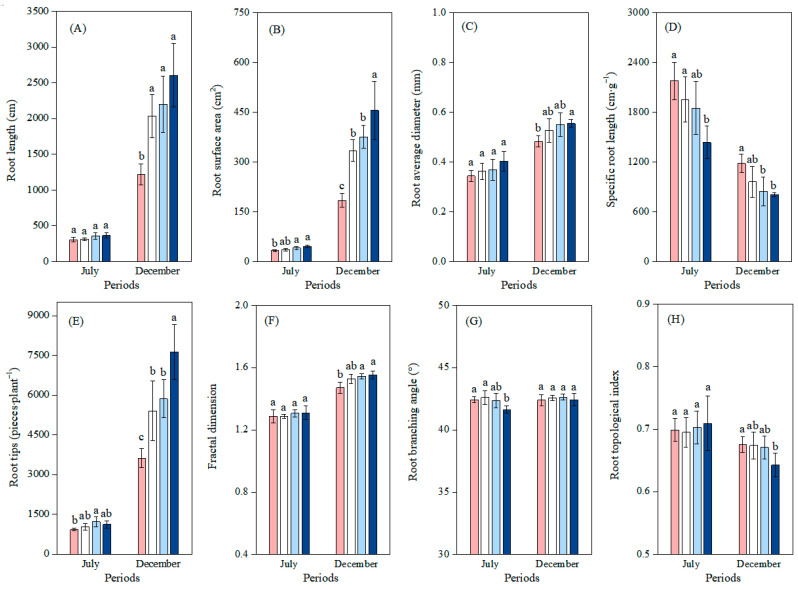
Effects of different soil phosphorus levels on the root morphology and architecture of moso bamboo seedlings. Note: a, b, c—different letters indicate significant differences among different treatments (*p* < 0.05). The values in the bar chart are the means and standard errors. Four phosphorus addition treatments: P1 (0 mg·kg^−1^), P2 (25 mg·kg^−1^), P3 (50 mg·kg^−1^), and P4 (100 mg·kg^−1^). Root morphological indices include (**A**): root length, (**B**): root surface area, (**C**): average root diameter, (**D**): specific root length. Root architectural indices include (**E**): root tip number, (**F**): fractal dimension, (**G**): root branching angle, (**H**): root topological index.

Regarding root morphology, during the first phase, there were no significant differences in RL and RD among different treatments (*p* > 0.05) (Figure 3). Compared with the P1 treatment, the P3 and P4 treatments significantly increased RSA by 17.0% (*p* < 0.05, 95% CI: 1.31, 15.13 cm^2^) and 19.3% (*p* < 0.01, 95% CI: 6.25, 20.06 cm^2^), respectively, and treatment P4 significantly decreased SRL by 34.1% (*p* < 0.01, 95% CI: −1209.2, −276.4). In the second phase, compared with treatment P1, the P addition treatments (P2, P3, and P4) significantly increased RL (by 66.8%, 80.5%, and 113.8%, respectively) and RSA (by 81.0%, 103.6%, and 146.5%, respectively) (*p* < 0.01). With increasing P addition, RD exhibited an increasing trend, while SRL showed a decreasing trend. Compared with treatment P1, the P4 treatment significantly increased RD by 14.8% (*p* < 0.05, 95% CI: 0.016, 0.128 mm), and the P3 and P4 treatments significantly decreased SRL (by 28.6% and 31.7%, respectively) (*p* < 0.05) (Figure 3).

Regarding root architecture, during the first phase, there were no significant differences in FD and TI among different treatments (*p* > 0.05) (Figure 3). Compared with P1 treatment, the P3 treatment significantly increased RT by 31.9% (*p* < 0.05), and P4 treatment significantly decreased RBA by 1.9% (*p* < 0.05, 95% CI: 0.195, 1.392°). In the second phase, compared with P1 treatment, the P2, P3, and P4 treatments significantly increased RT (by 49.1%, 62.8%, and 110.7%, respectively) (*p* < 0.05). The P3 and P4 treatments significantly increased FD (by 5.0% and 5.6%, respectively) (*p* < 0.05), and P4 treatment significantly decreased TI by 4.8% (95% CI: −0.060, −0.047) (*p* < 0.05).

**Figure 4:**
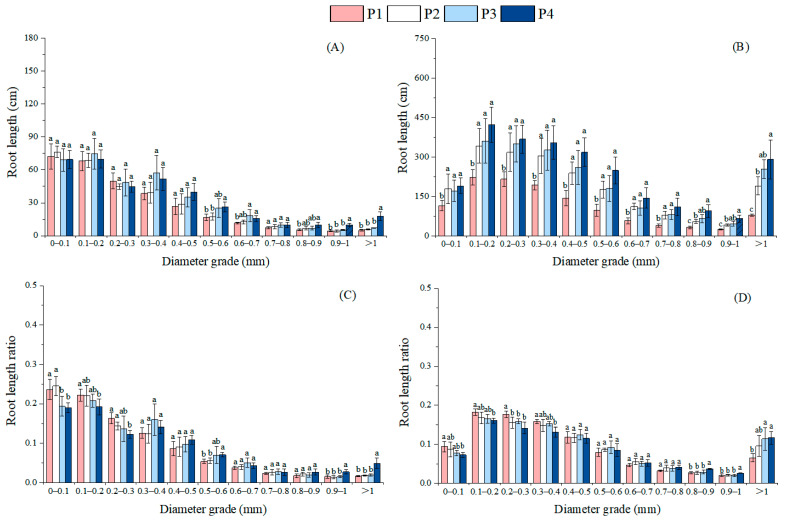
Effects of different soil phosphorus levels on the root length and root length ratio of roots with different diameter grades of moso bamboo seedlings. Note: a, b, c—different letters indicate significant differences among different treatments (*p* < 0.05). The values in the bar chart are the means and standard errors. Four phosphorus addition treatments: P1 (0 mg·kg^−1^), P2 (25 mg·kg^−1^), P3 (50 mg·kg^−1^), and P4 (100 mg·kg^−1^). (**A**): Root length results from July sampling, (**B**): root length results from December sampling, (**C**): root length ratio results from July sampling; (**D**): root length ratio results from December sampling.

In the first phase, the P4 treatment significantly decreased the RLR of the 0–0.3 mm root diameter grades compared to P1 (*p* < 0.01) (Figure 4). By contrast, P4 significantly increased the RLRs of the 0.9–1 mm root diameter grades by 83.8% (*p* < 0.01, 95% CI: 0.005, 0.020) and >1 mm root diameter grades by 194.4% (*p* < 0.01, 95% CI: 0.021, 0.05) relative to P1 treatment. During the second phase, increasing soil P levels induced a decreasing trend in RLRs for the 0–0.1 mm, 0.1–0.2 mm, 0.2–0.3 mm, and 0.3–0.4 mm root diameter grades, while the RLRs for the 0.7–0.8 mm, 0.8–0.9 mm, 0.9–1 mm, and >1 mm root diameter grades exhibited an increasing trend. Notably, P4 treatment significantly elevated the RLR in the >1 mm root diameter grades by 78.4% (*p* < 0.05, 95% CI: 0.005, 0.098) compared to P1 treatment. Furthermore, the RLRs in the 0.8–0.9 mm and 0.9–1 mm grades under P4 were significantly higher than those under P1, P2, and P3 treatments (*p* < 0.05).

Overall, compared with P addition treatments, moso bamboo under low-P treatment (P1) exhibited a higher SRL, RLR of finer roots, RBA, and TI, indicating that moso bamboo forms roots with lower construction costs, shallower root architecture, and greater capacity for rapid topsoil space occupation to adapt to low P. P addition reversed this stress-induced root morphological and architectural plasticity, which supports Hypothesis 1.

### 3.3. Effects of Different Soil Phosphorus Levels on the Nutrient Elements and Non-Structural Carbohydrates of Moso Bamboo Seedlings

In the first phase, the P content in roots, stems, and leaves increased gradually with elevated soil P levels (Table 3). The P contents in stems and leaves under P2, P3, and P4 treatments were significantly higher than those under P1 (*p* < 0.05). Compared with P1 treatment, the P2, P3, and P4 treatments significantly increased the leaf P content by 26.6%, 35.4%, and 51.8% (*p* < 0.05), respectively, and increased the stem P content by 17.8%, 26.1%, and 33.3% (*p* < 0.05), respectively. Compared with P1 treatment, the P3 and P4 treatments significantly increased the root P content by 13.4% (*p* < 0.05, 95% CI: 0.011, 0.175 mg·g^−1^) and 17.1% (*p* < 0.01, 95% CI: 0.037, 0.201 mg·g^−1^), respectively. Surprisingly, no significant difference was observed in the root P content between P1 and P2 treatments (*p* > 0.05). Compared with P1 treatment, the P3 and P4 treatments significantly increased the leaf N content by 15.8% (*p* < 0.01, 95% CI: 1.755, 5.165 mg·g^−1^) and 11.8% (*p* < 0.01, 95% CI: 0.895, 4.304 mg·g^−1^) and increased the stem N content by 16.7% (*p* < 0.01, 95% CI: 0.396, 2.072 mg·g^−1^) and 12.1% (*p* < 0.05, 95% CI: 0.054, 1.730 mg·g^−1^). No significant differences in the root N content were detected across the four treatments (p > 0.05). The N/P ratios in roots, stems, and leaves under P4 treatment were significantly lower than those under P1 treatment (*p* < 0.05). Notably, the leaf N/P ratio exhibited the steepest decline (by 26.2%).

In the second phase, the P contents in roots, stems, and leaves exhibited a trend analogous to that in the first phase (Table 3). A notable distinction was that, in the second phase, the root P content under the P2 treatment was significantly higher than that under the P1 treatment (*p* < 0.05), whereas no significant difference was observed between P1 and P2 in root P content during the first phase (*p* > 0.05). Across all P addition treatments (P2, P3, P4), the P contents in roots, stems, and leaves were significantly greater than those under the P1 treatment (*p* < 0.05).

In the first phase, with increasing soil P addition amounts, the contents of SS and starch in roots, stems, and leaves all exhibited a gradual upward trend, while the SS/ST ratio in roots and leaves gradually decreased (Table 4). Compared with P1 treatment, the P3 and P4 treatments significantly increased the SS content in leaves by 15.1% (*p* < 0.05, 95% CI: 0.068%, 2.564%) and 17.7% (*p* < 0.05, 95% CI: 0.288%, 2.784%). Compared with P1 treatment, the P3 and P4 treatments significantly increased the SS content in stems by 24.7% (*p* < 0.05, 95% CI: 0.927%, 3.117%) and 34.8% (*p* < 0.05, 95% CI: 1.762%, 3.951%), respectively. Compared with P1 treatment, P4 treatment significantly increased the SS content in roots by 15.9% (*p* < 0.05, 95% CI: 0.249%, 1.875%). There were no significant differences in the SS contents of leaves and stems between P1 and P2 treatments (*p* > 0.05), and no significant differences in the root SS content were observed among the P1, P2, and P3 treatments (*p* > 0.05). By contrast, the ST contents in the roots, stems, and leaves followed a significant increasing trend across the P1 < P2 < P4 gradient (*p* < 0.05), with P4 exhibiting the highest values. Compared with P1 treatment, the P2, P3, and P4 treatments significantly increased SS/ST in the leaves and roots (*p* < 0.05). In the second phase, the content and ratio of NSCs in roots, stems, and leaves exhibited a trend analogous to those in the first phase. A key distinction was that the leaf SS content under P2 treatment was significantly higher than that under P1 (*p* < 0.05). Surprisingly, the difference in the SS content in roots between P1 and P2 remained non-significant (*p* > 0.05). With increasing soil P addition amounts, the SS/ST ratios in roots, stems, and leaves all exhibited a gradual decline. ST was more profoundly influenced by P addition amount changes compared to SS. For instance, in the second phase, the root, stem, and leaf ST contents under the P4 treatment increased by 201.6%, 67.2%, and 102.4%, respectively, compared to P1, whereas the SS contents increased by only 22.5%, 30.8%, and 31.8%, respectively.

Compared with P addition treatments, low P significantly reduced the contents of P and SS in stems and leaves but had no effect on their contents in roots, indicating that moso bamboo allocated more P and SS to roots under low-P conditions. This also supports Hypothesis 3.

### 3.4. Effects of Different Soil Phosphorus Levels on the Nutrient Accumulation of Moso Bamboo Seedlings

**Figure 5:**
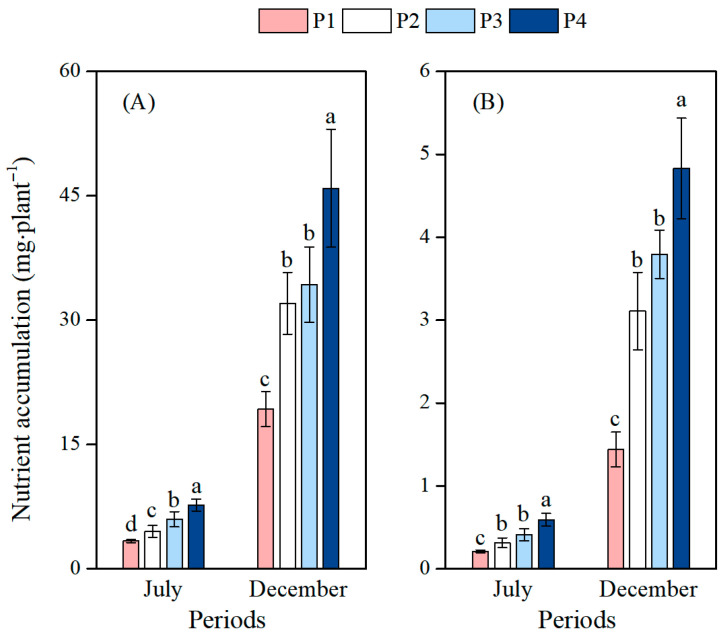
Effects of different soil phosphorus levels on the nutrient accumulation of moso bamboo seedlings. Note: a, b, c, d—different letters indicate significant differences among different treatments (*p* < 0.05). The values in the bar chart are the means and standard errors. (**A**): Nitrogen accumulation, (**B**): Phosphorus accumulation. Four phosphorus addition treatments: P1 (0 mg·kg^−1^), P2 (25 mg·kg^−1^), P3 (50 mg·kg^−1^), and P4 (100 mg·kg^−1^).

With increasing soil P addition amounts, the accumulation of both N and P exhibited a gradual upward trend (Figure 5). In the first phase, significant differences in total N accumulation were observed among the four treatments (*p* < 0.05). Compared with P1 treatment, the P2, P3, and P4 treatments significantly increased N accumulation by 35.0%, 79.4%, and 130.6%, respectively (*p* < 0.05), and increased P accumulation by 49.4%, 96.4%, and 181.5%, respectively (*p* < 0.05). In the second phase, compared with P1 treatment, the P2, P3, and P4 treatments significantly increased N accumulation by 66.3%, 78.0%, and 138.3%, respectively (*p* < 0.05), and increased P accumulation by 116.1%, 163.8%, and 235.8%, respectively (*p* < 0.01).

### 3.5. The Correlations Among Biomass, Root Morphology and Architecture, and Non-Structural Carbohydrates

**Figure 6:**
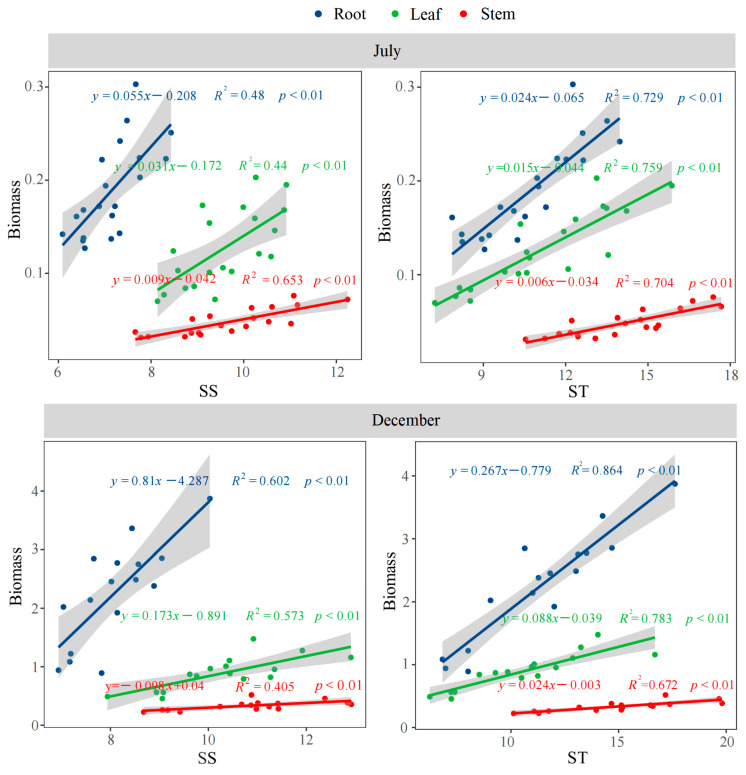
The correlations between the biomass and SS, and between the biomass and ST. SS: Soluble sugar, ST: Starch.

**Figure 7:**
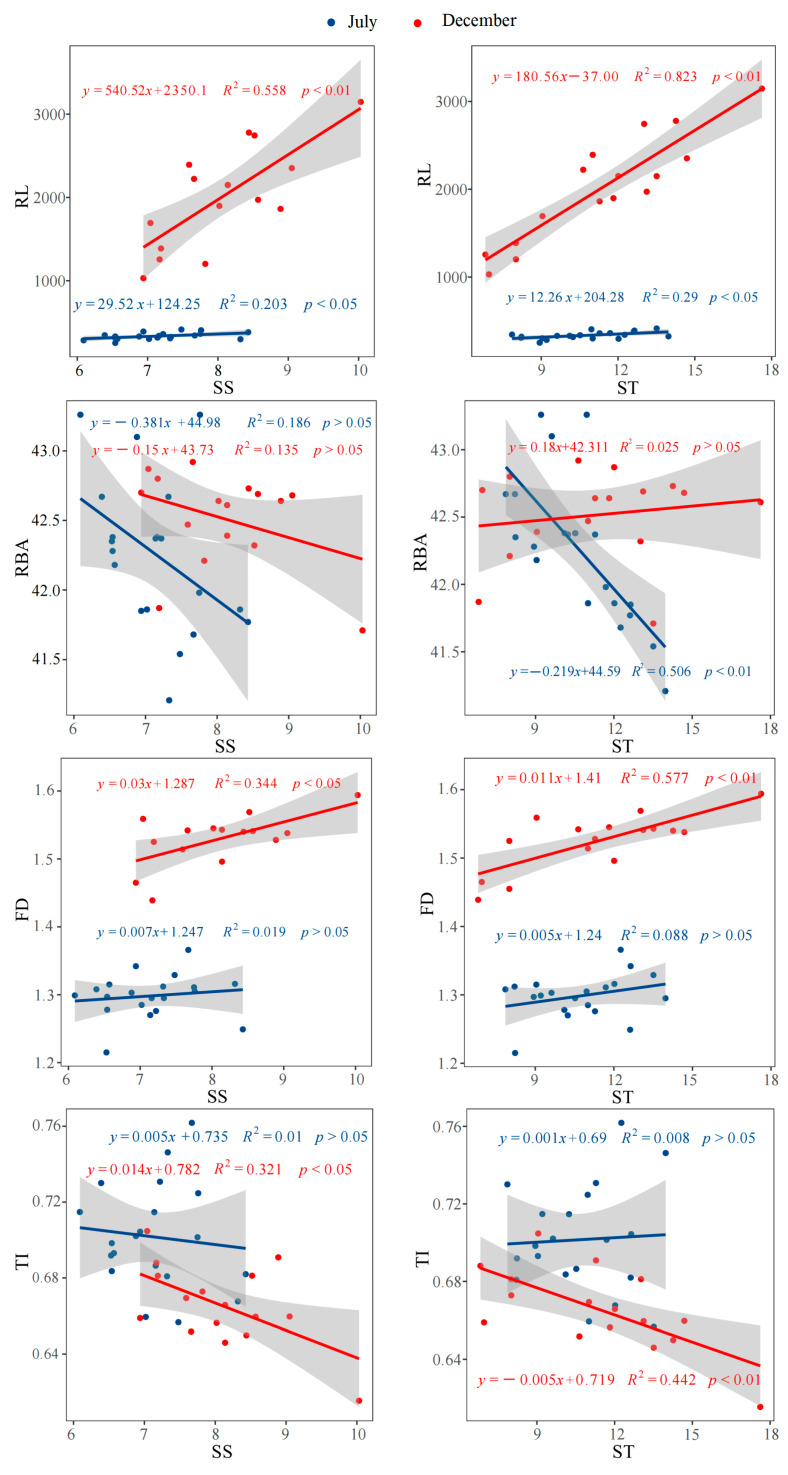
The correlations between root morphological and architectural indicators and SS, and between root morphological and architectural indicators and ST. SS: Soluble sugar, ST: Starch, RL: Root length, RBA: Root branching angle, FD: Fractal dimension, TI: Root topological index.

Across both periods, the biomass of leaves, stems, and roots exhibited highly significant positive correlations (*p* < 0.01) with both SS and ST (Figure 6). In July, RL showed a significant positive correlation (*p* < 0.05) with ST and SS. By December, RL exhibited extremely significant positive correlations (*p* < 0.01) with both ST and SS (Figure 7). Notably, RBA in July had an extremely significant negative correlation (*p* < 0.01) with ST (Figure 7). Additionally, FD in December was significantly positively correlated with SS (*p* < 0.05) and highly significantly positively correlated with ST (*p* < 0.01) (Figure 6). Conversely, TI in December showed significant negative correlations with both SS (*p* < 0.05) and ST (*p* < 0.01) (Figure 7).

## 4. Discussion

### 4.1. Responses of Biomass Allocation of Moso Bamboo Seedlings to Different Soil Phosphorus Levels

The regulatory capacity of plants’ biomass allocation strategies allows them to more effectively acquire resources under varying soil nutrient conditions, thereby sustaining growth, reproduction, and survival [31]. Hu’s study found that low P limits plant biomass accumulation by restricting chlorophyll synthesis and photosynthesis [32]. In this study, reduced biomass in roots, stems, and leaves of moso bamboo, along with decreased NSC contents under low-P conditions, corroborate this finding. Additionally, P addition significantly increased root, stem, and leaf biomass, confirming its role in alleviating low-P inhibition of moso bamboo growth. Zhang et al. reported a low-P-induced root biomass increase in maize, which contradicted our finding of reduced root biomass under low P [33]. This discrepancy may arise from species-specific differences in low-P adaptability and root P-activation capacity. For example, maize secretes more organic acids and phosphatases under low P to enhance rhizosphere P availability [34], whereas soybean increases root nodule number to compensate for P deficiency via enhanced N uptake [35]. By contrast, these adaptive mechanisms enable biomass accumulation under low P. Confounding variables also influence low-P responses—for instance, previous studies have reported contrasting maize biomass responses to low P in acidic vs. calcareous soils [36]. Although moso bamboo can enhance its adaptability to low P through root morphological plasticity, this strategy cannot eliminate the inhibitory effects of low P on its overall growth. However, during the first period, root biomass under the P1 treatment showed no significant difference compared to the P2 treatment. This indicates that low P had a relatively minor effect on root biomass accumulation in bamboo during the first period, whereas its impact on aboveground parts was more pronounced. Additionally, results from the root–shoot ratio showed that P addition significantly decreased this ratio; conversely, low-P stress significantly increased it. This is consistent with Zheng et al.’s study, which demonstrated that low P significantly reduced both shoot and root biomass in wheat while increasing the root–shoot ratio by 79.2% [37]. In the first growth stage, low-P treatment (P1) decreased biomass in roots, stems, and leaves by 13.4%, 17.2%, and 29.0% relative to P2, respectively. These results indicate that during the first half of the moso bamboo seedlings’ growing season, roots exhibited preferential growth under low-P stress. By contrast, in the second stage (December), low P significantly decreased the root–shoot ratio and caused biomass reductions of 51.5%, 20.3%, and 41.2% in roots, stems, and leaves, respectively. This suggests that during the second half of the growing season, the stems and leaves became the preferentially growing organs under low-P stress in moso bamboo. Previous studies have also revealed that the root–shoot ratio of moso bamboo exhibits divergent responses to P stress across seasons [13]. Based on these findings, a targeted fertilization strategy for moso bamboo cultivation could involve applying phosphate fertilizers to the root zone before July and foliar fertilizers after July, thereby achieving the objective of precision fertilization.

### 4.2. Responses of Root Morphology of Moso Bamboo Seedlings to Different Soil Phosphorus Levels

In this study, during the initial period, soil P levels exerted no significant effects on root length. However, as the growing season progressed, by December, the inhibitory effect of low P (P1) on root growth and the promotive effects of high P (P3 and P4) became more evident. This further corroborates that during the first half of the growing season, roots serve as the functional organs with preferential growth in moso bamboo under low-P stress. This preferential growth mechanism can, to a certain extent, mitigate the inhibitory effects of low P on root morphological development. However, studies on plants such as maize and *Fraxinus mandshurica* Rupr. have revealed that low P can promote the growth of lateral roots and the development of root hairs by facilitating the directional transport of auxin and gibberellin to the roots [38,39]. In this study, RD gradually decreased and SRL gradually increased as soil P levels declined, which aligns with the findings of most studies [15,40]. From the results of RLR, the reason why low P reduced the root diameter and increased SRL is that it promoted the growth of finer roots while restricting root thickening. This phenomenon may be attributed to the fact that low-P stress could induce smaller cortical cell size and tighter cellular arrangement in roots, while also inhibiting vascular bundle development to a certain degree [25]. In contrast, the effect of high P is opposite. Prioritizing the allocation of limited carbon to root elongation can, to a certain extent, sustain the growth of moso bamboo under low-P conditions. In contrast, radial growth of the roots requires a significant amount of carbon for cell wall thickening and cell enlargement, which is detrimental to the survival and growth of bamboo in low-P environments [41]. In this study, P addition increased root length by 66.8%, which was significantly higher than the 14.8% increase in root diameter, although differences between treatments were statistically significant. This indicates that the elongation growth of moso bamboo roots is more sensitive to phosphorus than radial growth. Kim et al. also found that increasing the P concentration in the growth substrate from 3 mg·L^−1^ to 20 mg·L^−1^ significantly increased the root length of *Lantana camara* L. but had no effect on root diameter [42].

### 4.3. Responses of Root Architecture of Moso Bamboo Seedlings to Different Soil Phosphorus Levels

Root architecture, which refers to the spatial configuration of a root system within the soil, has been demonstrated to play a crucial role in a plant’s acquisition of P [43]. In this study, low P significantly decreased the RT, suggesting that low P inhibits the formation of lateral root branches. This inhibitory effect becomes more pronounced in the second half of the growing season. This led to a significant decrease in the root system branching intensity (manifested as reduced FD). However, conflicting evidence from some studies suggests that low P promotes the formation and development of lateral roots and root hairs [44]. The different strategies of root growth in plants under low-P conditions may depend on whether plants possess genes that participate in regulating root growth. Some studies have found that PLDz genes are involved in regulating the different responses of primary roots and lateral roots to low P [45]. This is because under low-P conditions, these genes can promote the elongation of primary roots but inhibit the elongation of lateral roots. On the other hand, the LPR1 and LPR2 genes exhibit completely opposite functions [46]. The RBA and the root branching pattern (fractal dimension and topological structure) exhibited significant responses to P addition during the first half and second half of the growing seasons, respectively. During the first half of the growing season, the RBAs were larger under low-P conditions compared to high P, indicating that low P induces horizontal root expansion and the formation of relatively shallow root systems. The extension direction of plant roots (i.e., branching angle) under P deficiency also exhibits genotype dependency. For example, under low-P conditions, the proportion of shallow roots in sugarcane genotype ROC22 increased by 112% compared with that under normal P conditions, while no change or a decrease was observed in other sugarcane genotypes [47]. In the second half of the growing season, P addition increases the fractal dimension of the root system and decreases the topological index. Generally, it is believed that an increase in the TI leads to an increase in the construction cost of the root system, but it reduces the internal competition of the root system, which is beneficial for plants to enhance their adaptability to habitats with scarce water and nutrients [48,49].

### 4.4. Responses of Nutrient Element and Non-Structural Carbohydrate Allocation of Moso Bamboo Seedlings to Different Soil Phosphorus Levels

In this study, P addition significantly increased the P content in the aboveground parts (stems and leaves) of moso bamboo. Notably, during the first growth stage, no significant difference in the root P content was observed between the P1 and P2 treatments. This indicates that under low-P stress, moso bamboo tends to preferentially allocate P to the roots to maintain root physiological growth and nutrient absorption capacity. During the first half of the growing season, P supplementation also facilitated N uptake. However, the leaf N/P ratio results indicated that with increasing soil P content, the growth of moso bamboo transitioned from P-limited (N/P ratio > 16) to N-limited (N/P ratio < 14) [50]. During the second half of the growing season, the N content in roots, stems, and leaves exhibited a downward trend with increasing P levels. This phenomenon may be attributed to the elevated N demand of moso bamboo during the latter growth stage. Collectively, P supplementation promotes the synergistic uptake of N and P by moso bamboo; however, nitrogen fertilizer supplementation remains necessary to mitigate potential N limitation.

NSCs are important photosynthesis products supporting plant growth, metabolism, and a series of physiological activities [51]. Changes in the NSC concentration at growth parts can improve the flexibility of the plant growth in response to fluctuating environments [52]. In this study, P addition increased the contents of NSCs in roots, stems, and leaves, suggesting that P supplementation alleviates the constraints of low P availability on the production and accumulation of photosynthetic products. Compared to P2, low-P treatment increased the SS/ST in roots, stems, and leaves by 17.2%, 8.0%, and 28.0%, respectively. This indicated that moso bamboo maintained tissue growth under low-P conditions by converting ST into SS. Fu et al. found that low P enhances α-amylase activity in wheat, thereby promoting the conversion of ST to SS [53]. Previous studies showed that the sugar content was closely associated with plant lateral root density under varying soil P conditions, although it did not influence the length of the taproot [54]. SS showed a significant negative correlation with TI. The increase in SS accompanied by a decrease in TI further confirmed that SS promotion has a stronger effect on lateral root branching than on taproot growth. Additionally, studies showed that sugars could act as signaling molecules to induce the formation of cluster roots under low-P conditions, rather than serving solely as an energy source [55]. Throughout the growing season, low P had little effect on the SS content in roots but had a more pronounced impact on leaves and stems. This suggests that the directional transport of soluble sugar to roots to maintain their growth and physiological functions represents an important strategy for moso bamboo to adapt to low-P conditions. From the results of the correlation analysis, the adaptive changes in root morphology and architecture in response to increased soil P—such as the increase in RL and FD, and the decrease in TI—all benefit from the substantial accumulation of SS and ST in the roots. Previous results have also demonstrated that the extensive growth of moso bamboo roots in P-rich patches is accompanied by an increase in the content of photosynthetic products in the roots [15]. In this study, the change in root branching angle induced by P addition is also associated with starch in roots. This may be because P addition increases the starch content in roots, leading to enhanced root geotropism [56].

This study focused on data from moso bamboo seedlings. Given the differences in growth functions and environments between greenhouse-grown moso bamboo seedlings and adult bamboo in bamboo forests, the findings cannot be fully used as a basis for guiding P management in moso bamboo forests. Therefore, future research will focus on the response characteristics of mature bamboo in wild moso bamboo forests to P addition, in order to improve the understanding of the P response mechanism in moso bamboo.

## 5. Conclusions

In our study, moso bamboo seedlings adapted to soil P deficiency and carbon limitation caused by low P through root morphological plasticity, including reducing the root diameter, increasing the ratio of finer roots, and enhancing the specific root length. They also formed a herringbone-shaped shallow root architecture by decreasing the lateral root branching intensity and increasing root branching angles. Under low-P conditions, moso bamboo prioritized allocating photosynthates and P to roots, promoting the conversion of starch to soluble sugars to support root morphological and architectural plasticity and maintaining root growth and physiological functions. Additionally, during the first half of the growing season, low P imposed more significant constraints on shoot growth, leading to prioritized root growth; in the second half, root growth was more severely limited, with shoot growth becoming preferential. Although moso bamboo adapted to low P through morphological and architectural plasticity, its growth and nutrient accumulation remained significantly constrained. While simple P addition eliminated this constraint, it caused imbalances in nutrient element ratios, such as nitrogen limitation.

## Figures and Tables

**Table 1:** Soil nutrient contents and pH under different phosphorus addition treatments. HN: Hydrolyzable nitrogen, AP: available phosphorus.

Treatment	Sampling Time					
	July			December		
	pH	HN (mg·kg^−1^)	AP (mg·kg^−1^)	pH	HN (mg·kg^−1^)	AP (mg·kg^−1^)
P1	5.01 ± 0.09	79.9 ± 3.7	4.01 ± 0.20	4.95 ± 0.13	77.4 ± 3.6	3.72 ± 0.12
P2	5.11 ± 0.14	82.1 ± 3.0	8.99 ± 0.62	5.06 ± 0.15	77.5 ± 3.7	8.17 ± 0.47
P3	5.17 ± 0.08	82.5 ± 2.4	14.5 ± 0.84	5.08 ± 0.19	77.8 ± 2.9	13.7 ± 1.1
P4	5.09 ± 0.07	81.5 ± 4.0	29.9 ± 1.7	5.07 ± 0.11	76.5 ± 1.7	27.4 ± 2.0

**Table 2:** Abbreviations of all indicators.

Abbreviation	Full Form
RL	Root length
RSA	Root surface area
RD	Root average diameter
RLR	Root length ratio
Rl	Root length of each diameter grade
SRL	Specific root length
RT	Number of root tips
RBA	Root branching angle
FD	Fractal dimension
TI	Root topological index
N	Nitrogen
P	Phosphorus
NSCs	Non-structural carbohydrates
SS	Soluble sugar
ST	Starch

**Table 3:** Effect of different soil phosphorus levels on the nutrient element allocation of moso bamboo seedlings. Four phosphorus addition treatments: P1 (0 mg·kg^−1^), P2 (25 mg·kg^−1^), P3 (50 mg·kg^−1^), and P4 (100 mg·kg^−1^). P: phosphorus, N: nitrogen, N/P: the ratio of nitrogen to phosphorus content.

Period	Nutrient Content and Ratio	Treatment	Leaves	Stems	Roots
July	P (mg·g^−1^)	P1	1.17 ± 0.08 c	0.626 ± 0.034 b	0.696 ± 0.037 b
		P2	1.49 ± 0.11 b	0.738 ± 0.049 a	0.733 ± 0.064 ab
		P3	1.59 ± 0.08 b	0.790 ± 0.059 a	0.789 ± 0.036 a
		P4	1.78 ± 0.11 a	0.835 ± 0.072 a	0.815 ± 0.037 a
	N (mg·g^−1^)	P1	21.96 ±1.23 c	7.37 ± 0.55 b	9.85 ± 0.27 a
		P2	23.12 ± 1.11 bc	7.91 ± 0.50 ab	10.05 ± 0.38 a
		P3	25.42 ± 0.62 a	8.60 ± 0.46 a	10.59 ± 0.42 a
		P4	24.56 ± 0.66 ab	8.26 ± 0.31 a	10.38 ± 0.56 a
	N/P	P1	18.73 ±0.96 a	11.77 ± 0.72 a	14.18 ± 0.65 a
		P2	15.59 ± 0.58 b	10.73 ± 0.14 ab	13.77 ± 0.84 ab
		P3	16.00 ± 0.45 b	10.91 ± 0.37 ab	13.44 ± 0.74 ab
		P4	13.83 ± 0.96 c	9.96 ± 0.95 b	12.75 ± 0.59 b
December	P (mg·g^−1^)	P1	1.27 ± 0.09 b	0.602 ± 0.057 c	0.613 ± 0.066 b
		P2	1.49 ± 0.12 a	0.709 ± 0.045 b	0.736 ± 0.063 a
		P3	1.57 ± 0.05 a	0.740 ± 0.043 ab	0.810 ± 0.041 a
		P4	1.55 ± 0.13 a	0.820 ± 0.088 a	0.791 ± 0.047 a
	N (mg·g^−1^)	P1	16.35 ± 0.72 a	6.80 ± 0.41 a	8.84 ± 0.48 a
		P2	14.23 ± 1.06 b	6.49 ± 0.49 a	8.21 ± 0.42 a
		P3	12.78 ± 1.09 b	6.51 ± 0.47 a	7.78 ± 0.71 a
		P4	13.50 ± 0.95 b	6.48 ± 0.50 a	8.24 ± 0.31 a
	N/P	P1	12.91 ± 1.00 a	11.40 ± 1.56 a	14.57 ± 2.05 a
		P2	9.55 ± 0.50 b	9.20 ± 1.02 ab	11.25 ± 1.45 b
		P3	8.17 ± 0.76 b	8.83 ± 1.00 b	9.62 ± 0.92 b
		P4	8.80 ± 1.13 b	7.61 ± 0.60 b	10.34 ± 0.64 b

Note: a, b, c—different letters indicate significant differences among different treatments (*p* < 0.05). The values are the means and standard errors.

**Table 4:** Effect of different soil phosphorus levels on the non-structural carbohydrate allocation of moso bamboo seedlings. Four phosphorus addition treatments: P1 (0 mg·kg^−1^), P2 (25 mg·kg^−1^), P3 (50 mg·kg^−1^), and P4 (100 mg·kg^−1^).

Period	Non-Structural Carbohydrate Content and Ratio	Treatment	Leaves	Stems	Roots
July	Soluble sugar (%)	P1	8.69 ± 0.50 b	8.20 ± 0.56 c	6.67 ± 0.37 b
		P2	9.33 ± 0.87 ab	9.25 ± 0.36 bc	6.73 ± 0.42 b
		P3	10.01 ± 0.58 a	10.22 ± 0.65 ab	7.48 ± 0.56 ab
		P4	10.23 ± 0.74 a	10.68 ± 0.77 a	7.73 ± 0.42 a
	Starch (%)	P1	8.08 ± 0.53 c	11.81 ± 0.99 c	8.46 ± 0.51 c
		P2	10.38 ± 0.37 b	13.11 ± 1.22 bc	10.04 ± 0.68 b
		P3	12.06 ± 1.15 ab	14.69 ± 0.66 ab	11.48 ± 0.85 a
		P4	14.02 ± 1.11 a	16.53 ± 1.13 a	12.81 ± 0.93 a
	Sugar/starch	P1	1.077 ± 0.044 a	0.695 ± 0.035 a	0.791 ± 0.068 a
		P2	0.898 ± 0.069 b	0.710 ± 0.066 a	0.672 ± 0.033 b
		P3	0.834 ± 0.060 b	0.696 ± 0.035 a	0.654 ± 0.064 b
		P4	0.731 ± 0.045 c	0.670 ± 0.043 a	0.607 ± 0.065 b
December	Soluble sugar (%)	P1	8.76 ± 0.55 b	9.08 ± 0.31 b	7.28 ± 0.38 b
		P2	10.12 ± 0.52 a	10.83 ± 0.50 a	7.70 ± 0.50 b
		P3	10.78 ± 0.64 a	11.51 ± 0.95 a	8.41 ± 0.53 ab
		P4	11.55 ± 1.10 a	11.88 ± 0.89 a	8.92 ± 0.83 a
	Starch (%)	P1	7.02 ±0.53 c	11.07 ± 0.69 c	7.45 ± 0.65 c
		P2	9.85 ± 1.15 b	14.35 ± 0.96 b	10.97 ± 1.35 b
		P3	11.04 ± 0.93 b	15.73 ± 0.95 b	12.02 ± 1.24 b
		P4	14.21 ± 1.72 a	18.51 ± 1.42 a	15.02 ± 1.80 a
	Sugar/starch	P1	1.249 ± 0.028 a	0.822 ± 0.037 a	0.981 ± 0.064 a
		P2	1.034 ± 0.086 b	0.756 ± 0.036 ab	0.706 ± 0.048 b
		P3	0.979 ± 0.065 b	0.733 ± 0.055 b	0.704 ± 0.064 b
		P4	0.815 ± 0.059 c	0.642 ± 0.013 c	0.595 ± 0.020 c

Note: a, b, c—different letters indicate significant differences among different treatments (*p* < 0.05). The values are the means and standard errors.

## Data Availability

The data presented in this study are available on request from the corresponding author.
